# Increased intermediate M1-M2 macrophage polarization and improved cognition in mild cognitive impairment patients on ω-3 supplementation

**DOI:** 10.1096/fj.201600677RR

**Published:** 2016-09-27

**Authors:** Sam Famenini, Elizabeth A. Rigali, Henry M. Olivera-Perez, Johnny Dang, Michael To Chang, Ramesh Halder, Rammohan V. Rao, Matteo Pellegrini, Verna Porter, Dale Bredesen, Milan Fiala

**Affiliations:** * Department of Molecular, Cell, and Developmental Biology, University of California, Los Angeles, Los Angeles, California, USA;; † Buck Institute for Research on Aging, Novato, California, USA; and; ‡ Department of Neurology, University of California School of Medicine, Los Angeles, California; and; § Easton Laboratories for Neurodegenerative Disease, University of California School of Medicine, Los Angeles, California, USA

**Keywords:** ω-3 fatty acids, resolvin D1, amyloid-β phagocytosis, inflammation, ApoE genotype

## Abstract

Monocyte/macrophages of patients with mild cognitive impairment (MCI) and Alzheimer disease (AD) are defective in phagocytosis and degradation amyloid β_1–42_ (Aβ_1–42_), but are improved by ω-3 fatty acids (ω-3s). The hypothesis of this study was that active Aβ_1–42_ phagocytosis by macrophages prevents brain amyloidosis and thus maintains cognition. We studied the effects of self-supplementation with a drink with ω-3s, antioxidants, and resveratrol on Mini-Mental State Examination (MMSE) scores, macrophage M1M2 phenotype [the ratio of inflammatory cluster of differentiation (CD)54+CD80 and proresolution markers CD163+CD206], and Aβ_1–42_ phagocytosis in patients initially diagnosed as having MCI or subjective cognitive impairment (SCI). At baseline, the median MMSE score in patients in both the apolipoprotein E (ApoE) ε3/ε3 and ApoE ε3/ε4 groups was 26.0 and macrophage Aβ_1–42_ phagocytosis was defective. The MMSE rate of change increased in the ApoE ε3/ε3 group a median 2.2 points per year (*P* = 0.015 compared to 0) but did not change in the ApoE ε3/ε4 group (*P* = 0.014 between groups). In the ApoE ε3/ε3 group, all patients remained cognitively stable or improved; in the ApoE ε3/ε4 group, 1 recovered from dementia, but 3 lapsed into dementia. The macrophage phenotype polarized in patients bearing ApoE ε3/ε3 to an intermediate (green zone) M1-M2 type at the rate of 0.226 U/yr, whereas in patients bearing ApoE ε3/ε4, polarization was negative (*P* = 0.08 between groups). The baseline M1M2 type in the extreme M1 (red zone) or M2 (white zone) was unfavorable for cognitive outcome. Aβ_1–42_ phagocytosis increased in both ApoE groups (*P* = 0.03 in each groups). *In vitro*, the lipidic mediator resolvin D1 (RvD1) down regulated the M1 type in patients with ApoE ε3/ε3 but in some patients with ε3/ε4, paradoxically up-regulated the M1 type. Antioxidant/ω-3/resveratrol supplementation was associated with favorable immune and cognitive responses in ApoE ε3/ε3 and individual patients bearing ApoE ε3/ε4, and brings into personalized clinical practice the immune benefits expected from ω-3 mediators called resolvins. The validity of this study is limited by its small size and uncontrolled design.—Famenini, S., Rigali, E. A., Olivera-Perez, H. M., Dang, J., Chang, M T., Halder, R., Rao, R. V., Pellegrini, M., Porter, V., Bredesen, D., Fiala, M. Increased intermediate M1-M2 macrophage polarization and improved cognition in mild cognitive impairment patients on ω-3 supplementation.

Despite more than 200 clinical trials in Alzheimer disease (AD), there is no disease-modifying therapy for AD or for mild cognitive impairment (MCI). Actively or passively administered antibodies against amyloid β_1–42_ (Aβ_1–42_) have taken the lead in AD immunotherapy, but these approaches have so far encountered insurmountable difficulties caused by autoimmune manifestations and imaging abnormalities and have largely failed in clinical trials (1). We discovered the underlying immune defect of AD in phagocytosis of Aβ_1–42_ by macrophages in 2007 (2) and since then, have developed an immune approach with lipid mediators that regains functional Aβ_1–42_ phagocytosis (3). Innate immunity plays a bifunctional role in AD immunopathology: a negative role through IL-1-orchestrated inflammation (4) with activation of the membrane attack complex of complement C5b-9 (5, 6) and macrophage infiltration of the brain with disruption of the blood–brain barrier (7), and a positive role in uptake and clearance of Aβ_1–42_ by macrophages of healthy subjects (2). Aβ_1–42_ is the principal pathogenic molecule in the AD brain causing inflammation, neuronal apoptosis, disruption of neuronal connections, and congophilic angiopathy (8). Thus, appropriately regulating inflammation and increasing macrophage Aβ_1–42_ phagocytosis by ω-3 fatty acids (ω-3s) has a high therapeutic potential against MCI or subjective cognitive impairment (SCI).

The immunopathological mechanisms of failed Aβ_1–42_ clearance in patients with AD have not been reproduced in mouse models. In the mouse brain, Aβ_1–42_ clearance is attributable primarily to microglia (9). In the human brain, the macrophages have arguably a critical role in Aβ_1–42_ clearance, surmised from the defective macrophage phenotype of patients with AD (2) and disruption of the blood–brain barrier by apoptotic macrophages releasing Aβ_1–42_ into the vessel wall (10). In addition, the transcription of inflammatory genes in peripheral blood mononuclear cells (PBMCs) of patients with AD is deregulated either up or down in comparison to those in control subjects. Therefore, the increase of Aβ_1–42_ phagocytosis and modulation of inflammation by ω-3 docosahexaenoic acid (DHA), resolvin D1 (RvD1), and 1,25-dihydroxyvitamin D3 (1,25D3) (11) have therapeutic potential. The polyphenol resveratrol activates neuroprotective sirtuin-1 (Sirt1) and the genes for mitochondrial oxidative phosphorylation (12). Functioning macrophages could clear the AD brain, but patients have genetic heterogeneity in the apolipoprotein E (ApoE) gene and differences in macrophage responses. ApoE is a major lipid carrier in the brain. The ApoE ε4 allele is associated with late-onset AD through multiple mechanisms (13), including decreased Aβ_1–42_ clearance from the brain as observed in a mouse model (14), and functions as a transcription factor through binding to ∼1700 gene promoter regions associated with neuronal health (15). ApoE4 induces a proinflammatory state that is mediated, at least in part, by NF-κB, including a significant elevation of the proinflammatory cytokines IL6 and -8, which are associated with the pathologic changes found in AD (16). In addition, ApoE4 associates with soluble amyloid-precursor protein α (APPα) and reduces Sirt1 mRNA and protein expression (16, 15).

Brain-intrinsic mechanisms of DHA interacting with ApoE have been proposed in mouse models, including a change in the blood–brain barrier (17). However, these mechanisms do not address the immune defects of patients with MCI with specific changes in inflammatory activation, Aβ_1–42_ phagocytosis, and macrophage phenotype (see below). Health benefits of ω-3s have been extensively investigated and their effectiveness documented in rheumatoid arthritis (18). Epidemiologic studies of subjects at risk of dementia, with or without ω-3 intervention, have examined ω-3 blood levels and demonstrated that lower plasma concentrations of DHA are associated with cognitive decline (17). Seafood consumption correlates with lower AD neuropathology (19). In retrospective and some prospective intervention studies, ω-3s (20, 21) and vitamin D3 (22) had a positive effect on prevention of cognitive decline. The benefits of fish oil were stronger in non-ApoE ε4 subjects (23). In a randomized, placebo-controlled study, ω-3 supplementation reduced the rate of decline of the Mini–Mental State Examination (MMSE) score in comparison with a placebo, but the final MMSE score was below baseline (24). Thus, ω-3s, by themselves or as precursors of specialized proresolving mediators (25), appear appropriate for immunotherapy through brain-intrinsic and -extrinsic effects (26).

In this study, ω-3s were used in a combination with other active ingredients: antioxidants, vitamin D3, and resveratrol. This combination approach is in line with successful combination therapies for AIDS and tuberculosis, as well as with the metabolic enhancement of MCI therapy (27, 28). In a previous study, the Smartfish drink (Smartfish AS, Oslo, Norway) improved Aβ_1–42_ phagocytosis, normalized inflammation, and increased the anti-inflammatory mediator RvD1 (3). RvD1 is produced endogenously in macrophages from ω-3s (29) (30) and stimulates phagocytosis of Aβ_1–42_ (11). RvD1 signals through the GPCRs ALX and GPR32 (31), and Aβ_1–42_ phagocytosis enhancement by RvD1 is inhibited by a blockade of GPR32 (11). In addition, RvD1 promotes the proresolution phenotype of microglia *via* STAT-6 and PPAR-γ signaling (32).

We examined the effects of ω-3 supplementation on macrophage phenotype, phagocytosis, and MMSE in patients with subjective and objective defects in memory. Macrophage phenotype is involved in the outcome of many diseases, tissue homeostasis, and resolution or nonresolution of inflammation (33). The M1 status is associated with inflammatory conditions and is mitigated by ω-3s (34). Alternative activation of macrophages to M2 status has a bifunctional role in diseases, such as asthma (35, 36) and pancreatic fibrosis (37). We speculate that the outcome of immune therapy for patients with MCI depends in part on modulating inflammatory M1 (red zone) or anti-inflammatory M2 (white zone) macrophages at baseline to a proresolution and prophagocytic intermediate M1-M2 (green zone) phenotype.

Given the failures of previous monotherapeutic regimens and the advantages of combination therapies (27), we provided all but 1 patient with a drink containing ω-3s in combination with antioxidants, vitamin D3, and resveratrol. This drink has been used with success in controlled trials of behavioral problems (38) and obesity management. Nonetheless, in comparison to other disorders, the investigation of patients with MCI has been complicated by their baseline heterogeneity (11, 39), in part related to ApoE genotype. The patients selected for the study qualified as having MCI or SCI on admission, according to cognitive testing and shared the universal defect in Aβ_1–42_ phagocytosis. Imaging later showed specific diagnosis in 2 patients as Lewy body disease and vascular dementia. Our study has relevance to independently living patients with memory problems, irrespective of their pathology or brain imaging results.

## MATERIALS AND METHODS

### Study design and population

We performed an observational study by immune and cognitive tests of 18 independently living patients taking voluntary supplementation with the Smartfish ω-3 drink. Fifteen patients had the initial diagnosis of MCI by the criteria of Petersen *et al*. (40) and 3 patients the diagnosis of SCI, according to subjective complaints and subtle cognitive defects (41). Eleven patients (5 ApoE ε3/ε3 and 6 ApoE ε3/ε4) were followed by M1M2 testing in 90 visits. Cognition was tested by MMSE administered by the investigator. All subjects signed an informed consent approved by the University of California, Los Angeles Institutional Review Board.

### ω-3 nutritional supplementation

The patients took voluntary daily supplementation with the ω-3 drink (Smartfish AS) [a 200-ml emulsion containing 1000 mg DHA and 1000 mg eicosapentaenoic acid (EPA), both Pure arctic 360; Denomega, Oslo, Norway] protected against oxidation through nano-sized, stabilized emulsion droplets; 5 g whey protein (Lacprodan DI-7017; Arla Foods; Aarhus, Denmark), 0.2 g lactose; 18 g carbohydrate from pomegranate and chokeberry juice; 10 μg vitamin D3 stabilized with tocopherol (DSM Nutritional Products, Basel, Switzerland); and 150 mg resveratrol (resVida, DSM). One patient had been taking a softgel ω-3 capsule (300 mg ω-3) per day (Nature’s Bounty, Bohemia, NY, USA).

### Isolation of PBMCs and macrophage cultures

PBMCs were isolated from diluted heparin-anticoagulated blood by the Ficoll-Hypaque (Sigma-Aldrich, St. Louis, MO, USA) gradient method at 3000 rpm centrifugation for 20 min at room temperature. The mononuclear fraction was collected and washed 2 times with PBS, and the cells were resuspended with Iscove’s modified Dulbecco’s medium (IMDM). Macrophages were differentiated from 50,000 mononuclear cells by culture in IMDM with 10% autologous serum for 8–12 d in 8-well culture plates (Discovery Labware; BD Biosciences, Bedford, MA, USA).

### Macrophage phenotyping

Macrophages in 8-well chambers were washed with PBS, fixed with 4% paraformaldehyde, permeabilized with 0.2% Triton X-100, and stained with antibodies against cluster of differentiation (CD)54 (intercellular adhesion molecule-1), CD80 (B7-1), CD163 (scavenger receptor), and CD206 (mannose receptor) (all from BioLegend, San Diego, CA, USA; 2.5 μg/ml in PBS containing 1% bovine serum albumin), followed by appropriate secondary Alexa 565 antibodies and phalloidin-FITC (Sigma-Aldrich). Other markers included arginase-1 for M2 macrophages, and NOS2, TNF-α, IL-1β, IL-6, and IL-12 for M1 macrophages. The preparations were examined with a BX60 microscope with a ×20 objective (Olympus, Melville, NY, USA). Three images were obtained in the order top, middle, and bottom of the dense macrophage growth in each well. The images were scanned with Image-Pro software (MediaCybernetics, Rockville, MD, USA). The M1-M2 type was calculated as the ratio of (CD54+CD80)/(CD163+CD206) in mean fluorescence intensity (MFI) units.

### Modulation of macrophage type *in vitro*

Macrophages were incubated for 18 h, with or without RvD1 or -3 (27 ng/ml; Cayman Chemicals, Ann Arbor, MI, USA) and were phenotyped with CD54, CD80, CD163, and CD206 before and after the treatment.

### Flow cytometric fluorescent Aβ phagocytosis assay by monocytes

PBMCs (0.5 × 10^6^) were suspended in IMDM with 10% autologous serum and were incubated with or without 2 µg/ml of fluorescent Aβ_1–42_, HiLyte Fluor 488 (Anaspec, Fremont, CA, USA) overnight at 37°C in a 5% CO_2_ incubator. The cells were washed 2 times with a fluorescence-activated cell sorting (FACS) buffer and then labeled for 30 min at 4°C with anti-CD14 PE (BD Biosciences). After incubation, the cells were washed 2 times with FACS buffer and fixed with 1% paraformaldehyde. Flow cytometry was performed on FACSCalibur (BD Biosciences). The data were analyzed with FlowJo software (Tree Star, Ashland, OR, USA) with a monocyte gating, based on forward and side scatter, and the results are expressed in MFI units.

### Restriction isotyping of human ApoE

ApoE genotyping to identify the ApoE alleles was performed as described elsewhere (16). The method involved using primer pairs to amplify the region set in the common sequence parts of ApoE isoforms. The amplified products were later digested with *Hha*I and subjected to electrophoresis on polyacrylamide gels. Each of the isoforms was distinguished by a unique combination of *Hha*I fragment sizes that enabled explicit typing of all homozygotic and heterozygotic combinations.

### Statistical methods

For a given subject and outcome, the MMSE and MFI rates of change were computed as rate of change per year = 12[(last value − first value)/*n* mo follow-up] from first to last measurement. The M1/M2 ratio was coded 1 if the value was between 1 and 4 (in the green zone) and was coded 0 if below 1 or greater than 4 (outside of the green zone). The rates of change of these 0, 1 values were then computed for each subject as above. Rates of change were summarized with their medians. The *P* values for comparing continuous variables between groups including rates were computed with the nonparametric Kruskal-Wallis test, because the data did not follow normal distribution. The nonparametric Wilcoxon signed rank test was used to compute *P* values for comparing median rates to 0. Computations were performed with SAS 9.4 and JMP 12.0.1 (SAS, Inc., Cary, NC, USA).

## RESULTS

### Design of the study and overall results

**Figure 1.**
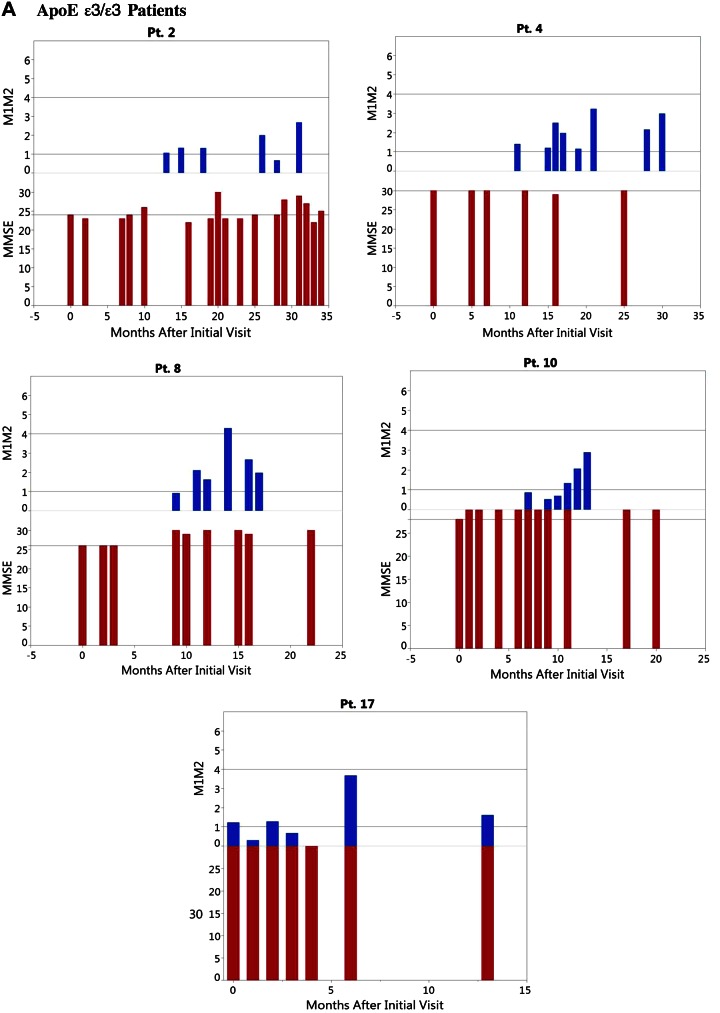

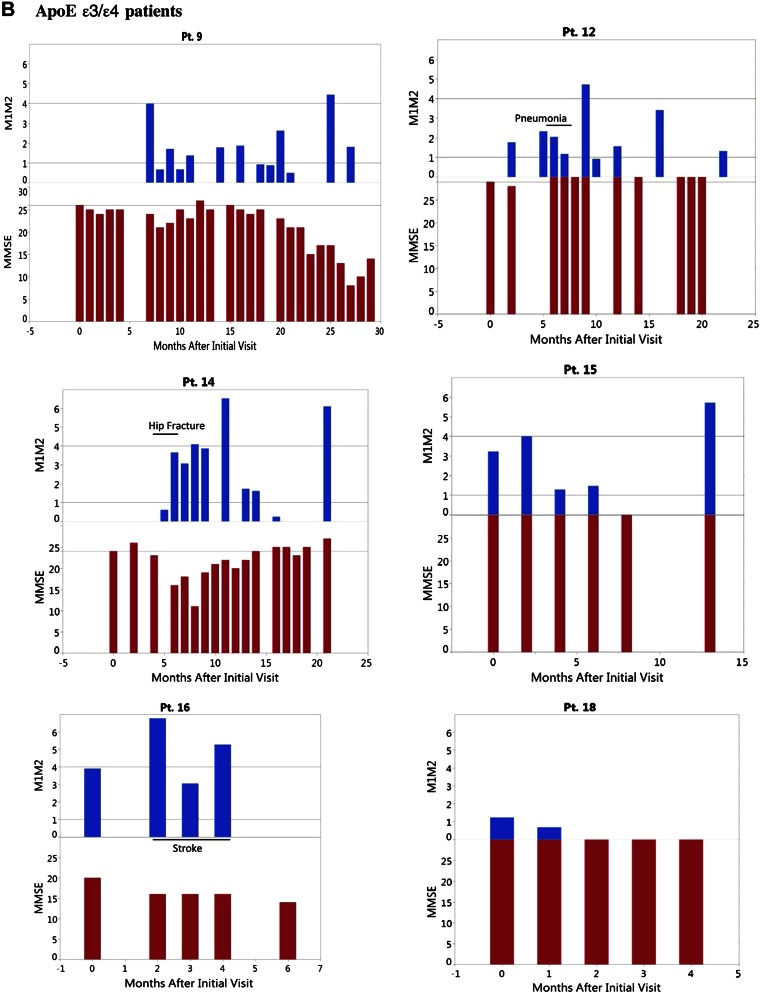
Immune and cognitive results in 5 patients with APO ε3/ε3 (*A*) and 6 with APO ε3/ε4 (*B*), according to the time after onset of ω-3 supplementation.

The study was an observation of patients with MCI on voluntary self-supplementation by ω-3s in the Smartfish drink. The patients were examined at each visit in 1–3 mo intervals by history and by testing macrophage type, level of Aβ_1–42_ phagocytosis, and MMSE score. The phenotype was scored according to the ratio of inflammatory over proresolution markers: (CD54+CD90)/(CD163+CD206). The M1M2 phenotype was considered to be noninflammatory (white zone) when the ratio was <1.0; inflammatory M1 (red zone) when the ratio was >4.0; and a proresolution M1-M2 (green zone associated with optimal Aβ_1–42_ phagocytosis) when the ratio was 1.0–4.0. The M1M2 results in the green zone were scored as 1 and in the white or red zone as 0. The results were analyzed according to the ApoE genotype (**Fig. 1** and **Table 1**).

**TABLE 1.** Immune (M1-M2) and cognitive (MMSE) results in patients bearing ApoE ε3/ε3 and ApoE ε3/ε4 MCI and taking the ω-3-supplemented drink

						*P*	
Variable	Group	*n*	Median	Min	Max	Between groups	Within group *vs.* 0
M1/M2							
Follow-up (mo)	ε3/ε3	5	6.0	5	15	0.5065	
	ε3/ε4	5	5.0	0	18		
Green zone							
Proportion							
60%	ε3/ε3	5	1.05	0.86	1.4		0.1877
70%	ε3/ε4	5	1.97	0.26	4.0		
Rate of change (yr^−1^)	ε3/ε3	5	0.23	−0.27	2.6		0.3750
	ε3/ε4	5	−0.06	−2.06	0.0	*0.0820*	0.1250
MFI, M1-M2							
Follow-up (mo)	ε3/ε3	9	3	1	22	0.5915	
	ε3/ε4	6	5	3	16		
Initial value	ε3/ε3	9	437	54.1	1,011	0.2875	
	ε3/ε4	6	414	399	433		
Rate of change (yr^−1^)	ε3/ε3	9	1041	−2,064	10,308	0.4795	*0.0391*
	ε3/ε4	6	2213	648	4,026		*0.0313*
MMSE							
Follow-up (mo)	ε3/ε3	9	11	1	29	0.7217	
	ε3/ε4	9	6	1	25		
Initial value	ε3/ε3	9	26.0	24	30	0.4416	
	ε3/ε4	9	26.0	20	30		
Rate of change (yr^−1^)	ε3/ε3	9	2.2	0.0	48.0	*0.0146*	*0.0156*
	ε3/ε4	9	0.0	−20.0	12.0		0.4063

Significant differences within and between groups are in italics. Rate of change (yr^−1^) into the green zone (M1/M2 ratio = 1–4) based on the coding as 1 (M1/M2 ratio = 1–4) ϴ (M1/M2 ratio <1 or >4).

The purpose of the study was to demonstrate the effect of ω-3s on 3 main objectives: *1*) MMSE rate of change (data available in 9 patients with ApoE ε3/ε3 and 9 with ApoE ε3/ε4); *2*) green zone M1-M2 phenotype change (data available in 5 patients with ApoE ε3/ε3 and 6 with ApoE ε3/ε4); and *3*) correlation between MMSE and M1-M2 phenotype (Table 1). The overall results were as presented below.

#### Objective 1

MMSE rate in the ApoE ε3/ε3 group (9 patients): median change by 0 points per year (*P* = 0.015 compared to 0); in the ApoE ε3/ε4 group (9 patients): median change by 0 points (*P* = 0.40 compared to 0); significant difference between groups (*P* = 0.014) (Table 1). In the ApoE ε3/ε3 group, 4 patients had cognitive improvement of 2 or more points, but in the ApoE ε3/ε4 group 4 patients showed severe cognitive loss at the last follow-up (**Table 2**).

**TABLE 2.** Individual immune and cognitive results in patients taking the ω-3-supplmented drink

				M1/M2		MFI		MMSE	
Patient	Age/sex	Diagnosis	Time in study (mo)	Initial visit	Final visit	Initial visit	Final visit	Initial visit	Final visit
ApoE ε3/ε3									
2	81/M	MCI	27	1.06	0.70	54	1186	24	29
4	78/M	Pre-MCI	21	1.40	3.23	377	1768	30	30
8	66/F	MCI	16	0.91	1.97	437	1825	26	29
10	54/F	MCI	19	0.86	2.88	679	1968	28	30
17	53/F	MCI	8	1.05	3.67	1011	1101	28	30
20	80/M	MCI	9			298	1157	26	30
21	70/M	MCI	4			437	1339	26	26
22	82/F	MCI	3			22	736	28	29
Mean	70		13.38	1.06	2.49	414.3	1385.0	27.0	29.1
sd				0.21	1.18	322.0	426.1	1.85	1.36
ApoE ε3/ε4									
3	40/M	MCI	10	0.26	1.06	399	1466	24	21
9	79/M	MCI	19	3.99	4.44	414	1880	26	17
12	76/M	MCI	17	1.76	3.41	1280.0	3267	29	30
14	90/F	MCI	17	0.61	0.25	400.0	1726	25	23
15	60/M	Pre-MCI	9	3.20	1.46	419.0	743	30	30
16	81/M	MCI-AD	10	4.03	5.28	433.0	1775	20	14
18	81/F	Pre-MCI	4	1.22	0.67			30	30
19	63/F	MCI	11	2.17		679	1616	26	30
23	88/F	MCI	8			414.0	1315.0	25	21
Mean	74		11.9	2.15	2.37	554.8	1723.5	26.1	24.0
sd				1.46	1.99	307.4	718.0	3.22	6.24

M1-M2 phenotype was tested by immunofluorescence microscopy, MFI by flow cytometry, and cognition by MMSE testing.

#### Objective 2

Median M1-M2 green zone rate change in the ApoE ε3/ε3 group was 0.226 per year (*P* = 0.375 compared to 0) and in the ApoE ε3/ε4 group median green zone change was −0.063 (*P* = 0.125 compared to 0); significant difference between groups, *P* = 0.082 (Table 1).

#### Objective 3

Correlation between MMSE and macrophage M1M2 phenotype: Spearman correlation in the ApoE ε3/ε3 group = 0.200 (*P* = 0.747) and in the ApoE ε3/ε4 group, Spearman correlation = 0.771 (*P* = 0.072); Spearman correlation of all patients **=** 0.483 (*P* = 0.132).

### On ω-3 supplementation ApoE ε3/ε3 macrophages polarize to the intermediate M1-M2 phenotype, whereas ApoE ε3/ε4 macrophages show irregular polarization

At baseline, patients bearing ApoE ε3/ε3 had low M1/M2 ratio (median M1/M2 ratio = 1.06), whereas patients with ApoE ε3/ε4 had higher M1/M2 ratio (median M1/M2 ratio = 1.97) (Tables 1 and **2**). Macrophages of patients bearing ApoE ε3/ε3 increased at the median M1-M2 rate of 0.226 U/yr (*P* = 0.375 compared to 0), and macrophages of patients with ApoE ε3/ε4 had negative M1-M2 polarization of −0.063 (*P* = 0.125 compared to 0). The significance of the M1-M2 rate change difference between the ApoE groups was (*P* = 0.082) (Table 1). The irregularities of patients with ApoE ε3/ε4 were related in part to intercurrent health problems and lack of ω-3 supplementation (Fig. 1). In patients with ApoE ε3/ε4, the baseline M1M2 type was in the red zone in 3 patients and increased further (4.4, 5.7, 5.28) and in 1 patient was in the white zone type and decreased further (from 0.61 to 0.25). All of these 4 patients had poor cognitive outcome (Table 2).

**Figure 2.**
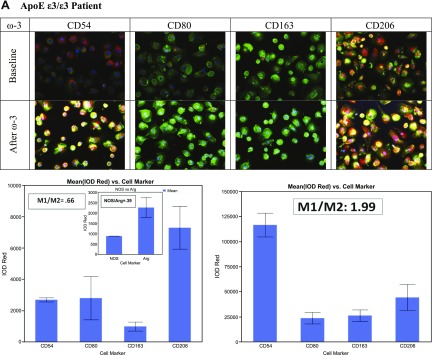

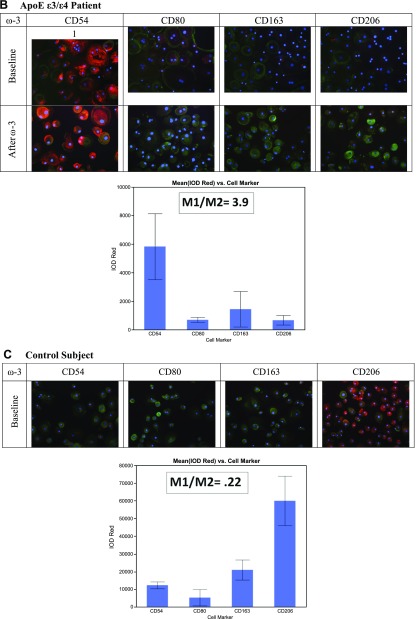
Macrophage type at baseline and after ω-3 supplementation. *A*) Polarization in patients with ApoE ε3/ε3 from baseline M2 to M1-M2 after ω-3 supplementation. *B*) Polarization in patients with ApoE ε3/ε4 from baseline M1 to greater M1 type after ω-3. *C*) Control subject M2 type at baseline (no ω-3 supplementation).

**Figure 3.**
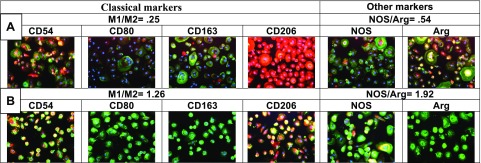
Classic and other markers of M2 macrophages (*A*) and M1-M2 macrophages (*B*).

An example of M1M2 polarization in the ApoE ε3/ε3 group (Fig. *2A*) shows the M1/M2 ratio increasing from baseline 0.66 to 1.99 (this patient had a baseline NOS/arginase ratio of 0.39). An example is shown where the ratio increased from 3.9 to 5.27 (Fig. 2*B*). A control age-matched subject had M2 type (ratio 0.22) macrophages (Fig. 2*C*) with excellent Aβ_1–42_ phagocytosis. Macrophage phenotype was confirmed by additional nonclassic markers, with the inflammatory marker NOS and the proresolution marker arginase (**Fig. 3**).

### Increased Aβ_1–42_ phagocytosis on ω-3 supplementation

On ω-3 supplementation, Aβ_1–42_ phagocytosis increased in both groups (Table 1). In the ApoE ε3/ε3 group, MFI increased with a median rate of change of 1041 MFI U/yr (*P* = 0.03 within group); in the ApoE ε3/ε4 group, with a median rate of change of 2213 MFI units per year (*P* = 0.03 within group; 0.47 between groups) (Table 1).

### Case histories of patients with ApoE ε3/ε3

#### Patient 2

A 79-yr-old man with diabetes mellitus under good control at baseline in 2014 with a history of memory problems in 2013. His initial MMSE was 23 and his diagnosis was MCI. He was in the study for 30 mo except during short periods of travel. He had prostatitis in July 2014. His MMSE continued to fluctuate, with a maximum MMSE score of 29. He was an avid bicyclist throughout the study until 2016 when he had a hip fracture after falling off his bicycle. Three months after his fall and hospitalization without the ω-3 drink, his MMSE dropped from 29 to 22.

#### Patient 4

A 77-yr-old active film producer had recurring diverticulitis and underwent a colectomy in March 2014. At baseline he complained of mental fog with poor memory for names and words, and forgetfulness of simple tasks. His MMSE was 30, and his diagnosis was SCI. He had been taking the drink for 27 mo, except during extensive periods of foreign travel. All his MMSE results were 30. His general condition improved after colectomy: his word memory improved, and he was active in his profession.

#### Patient 8

A 66-yr-old woman born in Afghanistan who received a PhD in international relations and was active as an advisor on women’s issues in Afghanistan. She developed problems with adding numbers in 2014. Her initial MMSE was 26, and her diagnosis was MCI. She took the drink for 22 mo with intermittent noncompliance because of her travels. Her MMSE improved from 26 to 30.

#### Patient 10

A 63-yr-old woman developed problems with remote memory and word- and concept-finding. Her initial MMSE was 28, and her diagnosis was SCI. She took the drink for 21 mo and increased complex carbohydrates and fish in her diet. Her remote memory improved and she developed a successful landscaping business.

#### Patient 17

A 54-yr-old woman with a strong family history of dementia developed problems with finding appropriate words and addition of numbers in March 2015. Her initial MMSE was 28, and her diagnosis was MCI. She had history of attention deficit hyperactivity disorder and Graves’ hyperthyroidism. She took a commercial fish oil preparation (not the Smartfish drink) for 8 mo. Her SPECTscan (single photon emission computed tomography) showed low activity overall. Her final MMSE was 30, but she continued to have word-finding difficulty.

### Case histories of patients with ApoE ε3/ε4

#### Patient 9

An 80-yr-old man, a retired civil engineer, took the drink for 22 mo. Before starting the study, he had a 2 yr history of memory and orientation problems. His first MMSE score was 26, and his diagnosis was MCI. His lowest MMSE was 6 in December 2015 when he was severely depressed and without supplementation. His MMSE improved to 23 in January 2016, but then his MMSE declined to 8, despite supervised supplementation. He had an increased homocysteine level of 15.6 μM (normal, 10.4–11.4 μM), normal C-reactive protein, a low DHEA level of 97 ng/ml (normal, 180–1250 ng/ml) and low testosterone of 0.7 pg/mo (normal, 30–135 pg/ml). Recently, his sensorium fluctuated, visual hallucinations were noted by his caregiver, and the fluorodeoxyglucose (FDG)–positron emission tomography/computed tomography (PET/CT) scan was consistent with dementia with Lewy bodies. He stopped taking the drink.

#### Patient 12

A 74-yr-old man, a retired attorney, had a 5 yr history of anxiety, poor sleep, poor memory, and word-finding difficulty. His MRI showed ventricular enlargement and temporal lobe atrophy; his FDG-PET showed reduced glucose utilization in the temporal lobes. His initial MMSE was 29, and his diagnosis was MCI. His lowest MMSE was 28 in August 2015 after having pneumonia in July 2015—final MMSE, 30. He took supplements for 20 mo. His homocysteine level was 16.4 μM (increased above normal) and DHEA was 1.56 ng/ml (below normal).

#### Patient 14

An 88-yr-old woman misplaced objects and had poor short-term memory, with an initial MMSE of 25; her diagnosis was MCI. She had taken the supplementation for 6 mo and then broke her hip in February 2015 and had a prolonged hospitalization without ω-3 supplementation until August 2015. Four months after the hip surgery, her MMSE declined to 11. She was restarted on the drink under supervision, and 4 mo later her MMSE increased to 25 and has remained between 24 and 27. She had low DHEA, but pregnenolone, progesterone, thyroid panel, and cortisol were normal. Her FDG-PET/CT showed hypometabolism of the posterior cingulate and parietotemporal cortex consistent with mild dementia. She did not take any AD drugs.

#### Patient 15

A 66-yr-old physician with MMSE 30 and depression at the start of study and took the supplemented drink. His initial MMSE was 30, and his diagnosis was SCI. His mood and office work improved while he took supplementation for 7 mo. He then failed to continue in the study for 6 mo, but returned complaining of failing recent memory, poor judgement, and depression. His MMSE remained at 30.

#### Patient 16

An 81-yr-old man was in the study for 11 mo. His initial diagnosis was MCI. He was taking supplements for 5 mo but had minor strokes that were diagnosed as multi-infarct dementia. His MMSE became unmeasurable, and he developed behavioral problems and was removed from the study by the caregiver.

### Polarization of macrophages by RvD1 *in vitro*

**Figure 4.**
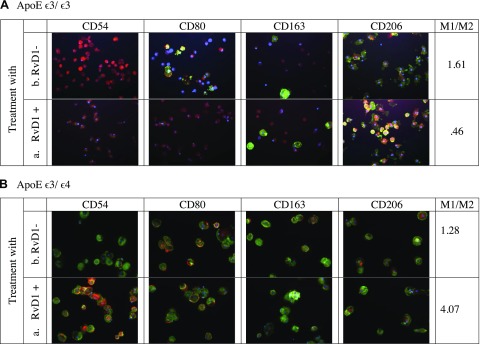
*In vitro* modulation of macrophage type by RvD1 stimulation. *A*) Patient with ApoE ε3/ε3, with (*a*) and without (*b*) RvD1. *B*) Patient with ApoE ε3/ε4, with (*a*) and without (*b*) RvD1.

In patients bearing ApoE ε3/ε3, *in vitro* treatment of macrophages by RvD1 reduced the M1/M2 ratio from 2.3 to 1.61, whereas in patients with ApoE ε3/ε4, this treatment increased the M1/M2 ratio from 1.89 to 2.35 (**Table 3**). An example of *in vitro* polarization by RvD1 in a patient with ApoE ε3/ε3 from 1.61 to 0.46 is shown **(****Fig. 4*A***). An example of idiosyncratic M1 polarization by RvD1 is shown where the ratio increased from 1.28 to 4.07 in a patient with ApoE ε3/ε4 (Fig. 4*B*).

**TABLE 3.** *In vitro* modulation of macrophage type by RvD1

Patient	Date	No stimulation	RvD1 stimulation
ApoE ε3/ε3			
2	5/31/2015	4.83	4.84
2	12/30/2015	1.99	0.89
2	2/21/2016	0.66	0.25
8	9/16/2015	1.97	1.27
10	5/31/2015	2.06	0.82
Mean		2.30	1.61
se		0.68	0.82
ApoE ε3/ε4			
12	9/30/2015	1.55	0.89
12	7/19/2016	1.31	2.58
14	5/31/2015	4.09	8.57
14	9/16/2015	1.76	1.66
14	12/31/2015	1.61	0.46
14	2/22/2016	0.25	0.67
15	9/17/2015	1.28	3.35
15	6/30/2016	5.71	3.45
16	9/17/2015	1.28	3.35
18	4/13/2016	0.67	0.30
19	4/13/2016	2.17	1.79
19	4/13/2016	1.07	1.19
Mean		1.90	2.36
se		0.44	0.66

Data are the M1/M2 ratio, with and without RvD1 stimulation. Macrophage phenotype was determined by immunofluorescence staining of macrophage with antibodies to CD54, CD80, CD163, and CD206.

## DISCUSSION

Our observational study of patients with MCI examined the effects of ω-3, antioxidant, and resveratrol drink supplementation on innate immunity and cognitive status in relationship to the ApoE genotype. Clinically and statistically significant findings (but limited by the study design and low number of subjects) were: *1*) in patients with ApoE ε3/ε3, a small improvement of cognitive level: MMSE improved by 2.2 points, whereas patients with ApoE ε3/ε4 did not change; the change within the ApoE ε3/ε3 group and between the groups was significant (*P* = 0.01); *2*) M1M2 polarization of macrophages in patients with ApoE ε3/ε3 to an M1-M2 type of 0.226 compared to the ApoE ε3/ε4 group rate of −0.063 was nearly significant (*P* = 0.08); *3*) correlation between the M1M2 rate and MMSE neared significance in the ApoE ε3/ε4 group (Spearman coefficient = 0.771, *P* = 0.072). The baseline M1M2 type in the red or white zone was a poor prognostic sign for cognition. The outstanding clinical “pearls” include patient 14, who recovered from dementia (MMSE 11) to her previous functioning (MMSE between 23 and 27) and patient 2, with improved MMSE score after 27 mo (initial 24, final 29). These results are limited by the small sample size; lack of randomization design to ω-3s *vs*. a placebo; intermittent compliance with ω-3 supplementation due to travel by patients 2, 4, and 12; diagnostic heterogeneity at baseline: 3 patients with SCI in the ApoE ε3/ε3 group *vs*. 2 with SCI and 1 with MCI-AD in the ApoE ε3/ε4 group; and the definition of the green zone M1/M2 ratio as 1–4 based on the observed relation of optimal M1-M2 to optimal MFI.

Overall results suggest that ω-3, antioxidant, and resveratrol supplementation were associated with stabilization or improvement for as long as 3 yr in the cognition of patients bearing ApoE ε3/ε3. The progress of those with ApoE ε3/ε4 was interrupted by intercurrent conditions, lack of supplementation, or a new diagnosis (Lewy body disease and multi-infarct dementia). An example of intercurrent conditions (hip fracture and hospitalization off supplementation), which were repaired by renewal of supplementation, is patient 14, who had a positive dementia FDG scan and lapsed into the dementia stage after surgery, but recovered to her previous cognitive status after discharge from the hospital and renewal of ω-3 supplementation.

Most patients with ApoE ε3/ε3 had M2 type at baseline, whereas 4 with ApoE ε3/ε4 had either extreme M1 red zone or M2 white zone macrophages. In the patients with ApoE ε3/ε3, ω-3 supplementation was associated with an increased rate of macrophage polarization to the M1-M2 type. In patients with ApoE ε3/ε4, polarization was irregular and associated with more extreme M1 macrophage type and poor outcome. Supplementation with ω-3 was associated with a significant improvement of MMSE status in patients with ApoE ε3/ε3 whose rate was significantly greater than 0 and superior in comparison to patients with ApoE ε3/ε4 (*P* = 0.01). The inferior results in patients with ApoE ε3/ε4 may be related to the known negative effects of ApoE ε4, such as a gain of toxic function through Aβ_1–42_ aggregation and decreased clearance (14), inflammatory activation of macrophages (42), decreased lipid and glucose metabolism, and interference with neuronal signaling and mitochondrial function (15). As expected, *in vitro* stimulation by RvD1 decreased the M1/M2 ratio in patients with ApoE ε3/ε3, but this stimulation paradoxically increased the M1/M2 ratio in some patients with ApoE ε3/ε4. The results of ω-3s are limited to patients with MCI and SCI, as there were no cognitive benefits of ω-3 supplementation in patients with AD (3).

## CONCLUSIONS

Supplementation with ω-3s/antioxidant/resveratrol provided in a drink with ω-3s and resveratrol is significantly (*P* = 0.01) associated with improved MMSE status and nonsignificantly (*P* = 0.08) associated with polarization of macrophages to an intermediate prophagocytic M1-M2 type in patients with ApoE ε3/ε3 in comparison to those with ApoE ε3/ε4. Supplementation with ω-3 brings into personalized clinical practice the immune benefits expected from ω-3 mediators called resolvins, and the results suggest a relationship between the improvements of innate immunity and cognition. Although the validity of this study is limited due to its small size and uncontrolled design, the results suggest a new immunologic approach to the widespread public health problem of dementia. The benefits of ω-3 supplementation should be clarified in larger controlled studies.

## Abbreviations

1,25D3: 1,25-dihydroxyvitamin D3
ω-3: ω-3 fatty acid
Aβ_1–42_: amyloid β_1–42_
AD: Alzheimer’s disease
ApoE: apolipoprotein E
APP: amyloid-precursor protein
CD: cluster of differentiation
Cur: curcumin
DHA: docosahexaenoic acid
EPA: eicosapentaenoic acid
FACS: fluorescence-activated cell sorting
FDG: fluorodeoxyglucose
IMDM: Iscove's modified Dulbecco's medium
MCI: mild cognitive impairment
MFI: mean fluorescence intensity
MMSE: Mini–Mental State Examination
PET: positron emission tomography
PBMC: peripheral blood mononuclear cell
PPAR: peroxisome proliferator-activated receptor
Res: resveratrol
RvD: resolvin D
SCI: subjective cognitive impairment
SIRT: sirtuin
SPM: specialized proresolving mediator

## References

[1] McGeer P. L., McGeer E. G. (2015) Targeting microglia for the treatment of Alzheimer’s disease. Expert Opin. Ther. Targets 19, 497–506 25435348 10.1517/14728222.2014.988707

[2] Fiala M., Liu P. T., Espinosa-Jeffrey A., Rosenthal M. J., Bernard G., Ringman J. M., Sayre J., Zhang L., Zaghi J., Dejbakhsh S., Chiang B., Hui J., Mahanian M., Baghaee A., Hong P., Cashman J. (2007) Innate immunity and transcription of MGAT-III and Toll-like receptors in Alzheimer’s disease patients are improved by bisdemethoxycurcumin. Proc. Natl. Acad. Sci. USA 104, 12849–12854 17652175 10.1073/pnas.0701267104 PMC1937555

[3] Fiala M., Halder R. C., Sagong B., Ross O., Sayre J., Porter V., Bredesen D. E. (2015) ω-3 Supplementation increases amyloid-β phagocytosis and resolvin D1 in patients with minor cognitive impairment. FASEB J. 29, 2681–2689 25805829 10.1096/fj.14-264218

[4] Mrak R. E., Griffin W. S. (2001) Interleukin-1, neuroinflammation, and Alzheimer’s disease. Neurobiol. Aging 22, 903–908 11754997 10.1016/s0197-4580(01)00287-1

[5] McGeer P. L., Akiyama H., Itagaki S., McGeer E. G. (1989) Activation of the classical complement pathway in brain tissue of Alzheimer patients. Neurosci. Lett. 107, 341–346 2559373 10.1016/0304-3940(89)90843-4

[6] Rogers J., Schultz J., Brachova L., Lue L. F., Webster S., Bradt B., Cooper N. R., Moss D. E. (1992) Complement activation and beta-amyloid-mediated neurotoxicity in Alzheimer’s disease. Res. Immunol. 143, 624–630 1455054 10.1016/0923-2494(92)80046-n

[7] Fiala M., Liu Q. N., Sayre J., Pop V., Brahmandam V., Graves M. C., Vinters H. V. (2002) Cyclooxygenase-2-positive macrophages infiltrate the Alzheimer’s disease brain and damage the blood-brain barrier. Eur. J. Clin. Invest. 32, 360–371 12027877 10.1046/j.1365-2362.2002.00994.x

[8] Hardy J., Selkoe D. J. (2002) The amyloid hypothesis of Alzheimer’s disease: progress and problems on the road to therapeutics. Science 297, 353–356 12130773 10.1126/science.1072994

[9] ElAli A., Rivest S. (2016) Microglia in Alzheimer’s disease: a multifaceted relationship. Brain Behav. Immun. 55, 138–150 10.1016/j.bbi.2015.07.021 26254232

[10] Zaghi J., Goldenson B., Inayathullah M., Lossinsky A. S., Masoumi A., Avagyan H., Mahanian M., Bernas M., Weinand M., Rosenthal M. J., Espinosa-Jeffrey A., de Vellis J., Teplow D. B., Fiala M. (2009) Alzheimer disease macrophages shuttle amyloid-beta from neurons to vessels, contributing to amyloid angiopathy. Acta Neuropathol. 117, 111–124 19139910 10.1007/s00401-008-0481-0 PMC5650912

[11] Mizwicki M. T., Liu G., Fiala M., Magpantay L., Sayre J., Siani A., Mahanian M., Weitzman R., Hayden E. Y., Rosenthal M. J., Nemere I., Ringman J., Teplow D. B. (2013) 1α,25-Dihydroxyvitamin D3 and resolvin D1 retune the balance between amyloid-β phagocytosis and inflammation in Alzheimer’s disease patients. J. Alzheimers Dis. 34, 155–170 23186989 10.3233/JAD-121735 PMC4040018

[12] Lagouge M., Argmann C., Gerhart-Hines Z., Meziane H., Lerin C., Daussin F., Messadeq N., Milne J., Lambert P., Elliott P., Geny B., Laakso M., Puigserver P., Auwerx J. (2006) Resveratrol improves mitochondrial function and protects against metabolic disease by activating SIRT1 and PGC-1alpha. Cell 127, 1109–1122 17112576 10.1016/j.cell.2006.11.013

[13] Liu C. C., Kanekiyo T., Xu H., Bu G. (2013) Apolipoprotein E and Alzheimer disease: risk, mechanisms and therapy. Nat. Rev. Neurol. 9, 106–118 23296339 10.1038/nrneurol.2012.263 PMC3726719

[14] Castellano J. M., Kim J., Stewart F. R., Jiang H., DeMattos R. B., Patterson B. W., Fagan A. M., Morris J. C., Mawuenyega K. G., Cruchaga C., Goate A. M., Bales K. R., Paul S. M., Bateman R. J., Holtzman D. M. (2011) Human apoE isoforms differentially regulate brain amyloid-β peptide clearance. Sci. Transl. Med. 3, 89ra57 10.1126/scitranslmed.3002156 PMC3192364 21715678

[15] Theendakara V., Peters-Libeu C. A., Spilman P., Poksay K. S., Bredesen D. E., Rao R. V. (2016) Direct transcriptional effects of apolipoprotein E. J. Neurosci. 36, 685–700 26791201 10.1523/JNEUROSCI.3562-15.2016 PMC4719010

[16] Theendakara V., Patent A., Peters Libeu C. A., Philpot B., Flores S., Descamps O., Poksay K. S., Zhang Q., Cailing G., Hart M., John V., Rao R. V., Bredesen D. E. (2013) Neuroprotective Sirtuin ratio reversed by ApoE4. Proc. Natl. Acad. Sci. USA 110, 18303–18308 24145446 10.1073/pnas.1314145110 PMC3831497

[17] Salem N. Jr., Vandal M., Calon F. (2015) The benefit of docosahexaenoic acid for the adult brain in aging and dementia. Prostaglandins Leukot. Essent. Fatty Acids 92, 15–22 25457546 10.1016/j.plefa.2014.10.003

[18] Calder P. C. (2015) Marine omega-3 fatty acids and inflammatory processes: Effects, mechanisms and clinical relevance. Biochim. Biophys. Acta 1851, 469–484 25149823 10.1016/j.bbalip.2014.08.010

[19] Morris M. C., Brockman J., Schneider J. A., Wang Y., Bennett D. A., Tangney C. C., van de Rest O. (2016) Association of seafood consumption, brain mercury level, and APOE ε4 status with brain neuropathology in older adults. JAMA 315, 489–497 26836731 10.1001/jama.2015.19451 PMC5460535

[20] Jicha G. A., Markesbery W. R. (2010) Omega-3 fatty acids: potential role in the management of early Alzheimer’s disease. Clin. Interv. Aging 5, 45–61 20396634 10.2147/cia.s5231 PMC2854051

[21] Cederholm T., Salem N. Jr., Palmblad J. (2013) ω-3 fatty acids in the prevention of cognitive decline in humans. Adv. Nutr. 4, 672–676 24228198 10.3945/an.113.004556 PMC3823515

[22] Annweiler C., Llewellyn D. J., Beauchet O. (2013) Low serum vitamin D concentrations in Alzheimer’s disease: a systematic review and meta-analysis. J. Alzheimers Dis. 33, 659–674 23042216 10.3233/JAD-2012-121432

[23] Huang T. L., Zandi P. P., Tucker K. L., Fitzpatrick A. L., Kuller L. H., Fried L. P., Burke G. L., Carlson M. C. (2005) Benefits of fatty fish on dementia risk are stronger for those without APOE epsilon4. Neurology 65, 1409–1414 16275829 10.1212/01.wnl.0000183148.34197.2e

[24] Freund-Levi Y., Eriksdotter-Jönhagen M., Cederholm T., Basun H., Faxén-Irving G., Garlind A., Vedin I., Vessby B., Wahlund L. O., Palmblad J. (2006) Omega-3 fatty acid treatment in 174 patients with mild to moderate Alzheimer disease: OmegAD study: a randomized double-blind trial. Arch. Neurol. 63, 1402–1408 17030655 10.1001/archneur.63.10.1402

[25] Serhan C. N. (2014) Pro-resolving lipid mediators are leads for resolution physiology. Nature 510, 92–101 24899309 10.1038/nature13479 PMC4263681

[26] Fiala M., Terrando N., Dalli J. (2015) Specialized pro-resolving mediators from omega-3 fatty acids improve amyloid-β phagocytosis and regulate inflammation in patients with minor cognitive impairment. J. Alzheimers Dis. 48, 293–301 26401996 10.3233/JAD-150367

[27] Bredesen D. E. (2014) Reversal of cognitive decline: a novel therapeutic program. Aging (Albany, N.Y.) 6, 707–717 10.18632/aging.100690 PMC4221920 25324467

[28] Bredesen D. E., Amos E. C., Canick J., Ackerley M., Raji C., Fiala M., Ahdidan J. (2016) Reversal of cognitive decline in Alzheimer’s disease. Aging (Albany, N.Y.) 8, 1250–1258 10.18632/aging.100981 PMC4931830 27294343

[29] Serhan C. N., Chiang N., Dalli J. (2015) The resolution code of acute inflammation: Novel pro-resolving lipid mediators in resolution. Semin. Immunol. 27, 200–215 25857211 10.1016/j.smim.2015.03.004 PMC4515371

[30] Serhan C. N., Petasis N. A. (2011) Resolvins and protectins in inflammation resolution. Chem. Rev. 111, 5922–5943 21766791 10.1021/cr100396c PMC3192290

[31] Serhan C. N., Krishnamoorthy S., Recchiuti A., Chiang N. (2011) Novel anti-inflammatory--pro-resolving mediators and their receptors. Curr. Top. Med. Chem. 11, 629–647 21261595 10.2174/1568026611109060629 PMC3094721

[32] Li L., Wu Y., Wang Y., Wu J., Song L., Xian W., Yuan S., Pei L., Shang Y. (2014) Resolvin D1 promotes the interleukin-4-induced alternative activation in BV-2 microglial cells. J. Neuroinflammation 11, 72 24708771 10.1186/1742-2094-11-72 PMC3983859

[33] Sica A., Mantovani A. (2012) Macrophage plasticity and polarization: in vivo veritas. J. Clin. Invest. 122, 787–795 22378047 10.1172/JCI59643 PMC3287223

[34] Schif-Zuck S., Gross N., Assi S., Rostoker R., Serhan C. N., Ariel A. (2011) Saturated-efferocytosis generates pro-resolving CD11b low macrophages: modulation by resolvins and glucocorticoids. Eur. J. Immunol. 41, 366–379 21268007 10.1002/eji.201040801 PMC3082320

[35] Girodet P. O., Nguyen D., Mancini J. D., Hundal M., Zhou X., Israel E., Cernadas M. (2016) Alternative macrophage activation is increased in asthma [E-pub ahead of print]. Am. J. Respir. Cell Mol. Biol. doi: 0.1165/rcmb.2015-0295OC 10.1165/rcmb.2015-0295OC PMC5070104 27248771

[36] Croasdell A., Thatcher T. H., Kottmann R. M., Colas R. A., Dalli J., Serhan C. N., Sime P. J., Phipps R. P. (2015) Resolvins attenuate inflammation and promote resolution in cigarette smoke-exposed human macrophages. Am. J. Physiol. Lung Cell. Mol. Physiol. 309, L888–L901 26301452 10.1152/ajplung.00125.2015 PMC4609942

[37] Xue J., Schmidt S. V., Sander J., Draffehn A., Krebs W., Quester I., De Nardo D., Gohel T. D., Emde M., Schmidleithner L., Ganesan H., Nino-Castro A., Mallmann M. R., Labzin L., Theis H., Kraut M., Beyer M., Latz E., Freeman T. C., Ulas T., Schultze J. L. (2014) Transcriptome-based network analysis reveals a spectrum model of human macrophage activation. Immunity 40, 274–288 24530056 10.1016/j.immuni.2014.01.006 PMC3991396

[38] Raine A., Portnoy J., Liu J., Mahoomed T., Hibbeln J. R. (2015) Reduction in behavior problems with omega-3 supplementation in children aged 8-16 years: a randomized, double-blind, placebo-controlled, stratified, parallel-group trial. J. Child Psychol. Psychiatry 56, 509–520 25146492 10.1111/jcpp.12314 PMC4336833

[39] Mizwicki M. T., Menegaz D., Zhang J., Barrientos-Durán A., Tse S., Cashman J. R., Griffin P. R., Fiala M. (2012) Genomic and nongenomic signaling induced by 1α,25(OH)2-vitamin D3 promotes the recovery of amyloid-β phagocytosis by Alzheimer’s disease macrophages. J. Alzheimers Dis. 29, 51–62 22207005 10.3233/JAD-2012-110560

[40] Petersen R. C., Doody R., Kurz A., Mohs R. C., Morris J. C., Rabins P. V., Ritchie K., Rossor M., Thal L., Winblad B. (2001) Current concepts in mild cognitive impairment. Arch. Neurol. 58, 1985–1992 11735772 10.1001/archneur.58.12.1985

[41] Duara R., Loewenstein D. A., Greig M. T., Potter E., Barker W., Raj A., Schinka J., Borenstein A., Schoenberg M., Wu Y., Banko J., Potter H. (2011) Pre-MCI and MCI: neuropsychological, clinical, and imaging features and progression rates. Am. J. Geriatr. Psychiatry 19, 951–960 21422909 10.1097/JGP.0b013e3182107c69 PMC3175279

[42] Jofre-Monseny L., Loboda A., Wagner A. E., Huebbe P., Boesch-Saadatmandi C., Jozkowicz A., Minihane A. M., Dulak J., Rimbach G. (2007) Effects of apoE genotype on macrophage inflammation and heme oxygenase-1 expression. Biochem. Biophys. Res. Commun. 357, 319–324 17416347 10.1016/j.bbrc.2007.03.150 PMC2096715

